# FosA3 emerging in clinical carbapenemase-producing *C. freundii*

**DOI:** 10.3389/fcimb.2024.1447933

**Published:** 2024-08-06

**Authors:** Vittoria Mattioni Marchetti, Irene Venturelli, Tiziana Cassetti, Marianna Meschiari, Roberta Migliavacca, Ibrahim Bitar

**Affiliations:** ^1^ Microbiology and Clinical Microbiology Unit, Scienze Clinico, Chirurgiche, Diagnostiche, Pediatriche (SCCDP) Department, University of Pavia, Pavia, Italy; ^2^ Clinical Microbiology, Azienda Unità Sanitaria Locale (AUSL) Modena, Modena, Italy; ^3^ Infectious Diseases Clinic, Azienda Ospedaliera Universitaria (AOU) Policlinico di Modena, Modena, Italy; ^4^ I.R.C.C.S. Policlinico S. Matteo, Pavia, Italy; ^5^ Department of Microbiology, Faculty of Medicine, University Hospital in Pilsen, Charles University, Pilsen, Czechia; ^6^ Biomedical Center, Faculty of Medicine, Charles University, Pilsen, Czechia

**Keywords:** fosfomycin, *Citrobacter freundii*, carbapenemases, fosfomycin resistance, *fosA3* gene

## Abstract

Fosfomycin (FOS) is an effective antibiotic against multidrug-resistant *Enterobacterales*, but its effectiveness is reducing. Little is known on the current prevalence of FosA enzymes in low-risk pathogens, such as *Citrobacter freundii*. The aim of the study was the molecular characterization of a carbapenemase- and FosA-producing *C. freundii* collected in Italy. AK867, collected in 2023, showed an XDR profile, retaining susceptibility only to colistin. AK867 showed a FOS MIC >128 mg/L by ADM. Based on WGS, AK867 belonged to ST116 and owned a wide resistome, including *fosA3*, *bla*KPC-2, and *bla*VIM-1. *fosA3* was carried by a conjugative pKPC-CAV1312 plasmid of 320,480 bp, on a novel composite transposon (12,907 bp). FosA3 transposon shared similarities with other *fosA3*-harboring pKPC-CAV1312 plasmids among *Citrobacter* spp. We report the first case of FosA3 production in clinical carbapenemase-producing *C. freundii* ST116. The incidence of FosA3 enzymes is increasing among *Enterobacterales*, affecting even low-virulence pathogens, as *C. freundii*.

## 1 Introduction

Carbapenem-resistant *Enterobacteriaceae* (CRE) infections, which present a considerable challenge for clinicians, are an increasing global threat. Currently, combination therapy based on carbapenem and fosfomycin is a valid option for the treatment of CRE infections (Bakthavatchalam et al., 2020).

Fosfomycin (FOS) is a phosphoric acid derivate, active against both Gram-negative and Gram-positive bacteria, which regained clinical interest in the last 20 years as a valid candidate in the treatment of multidrug-resistant (MDR) infections (Dijkmans et al., 2017). Currently, FOS is indicated for the treatment of uncomplicated UTIs, whereas the parenteral FOS has been used in case of systemic infections caused by MDR organisms (Falagas et al., 2009). FOS used in combination therapy is usually associated with good clinical outcome and bacteriological cure (Shiju et al., 2020). However, in recent years, an increased rate of resistant bacteria has been reported globally, mainly due to FOS-modifying enzymes (such as FosA).

As of February 2024, 10 *fosA* variants have been reported in the members of *Enterobacterales*. Plasmid-mediated dissemination of *fosA*-like genes is recognized as a worrying new challenge for the public health; *fosA3* is the most widespread variant, with endemic cases reported from both veterinary and clinical settings in China (Singkham-In et al., 2020; Zhang et al., 2020; Wang et al., 2021; Zou et al., 2021; Mattioni Marchetti et al., 2023a).

*Citrobacter* spp. are considered as low-risk pathogens, yet can act as silent reservoirs for relevant resistance genes, especially in case of *Citrobacter freundii* (Bitar et al., 2019). Recent evidence has suggested that the rate of infections caused by carbapenemase-producing *Citrobacter* spp. is increasing, with relevant reports among Mediterranean countries (Yao et al., 2021; Nobrega et al., 2023). In this scenario, high FOS MICs may further impair antibiotic effectiveness. The co-occurrence of carbapenemases and FosA in *Citrobacter* spp. is scarcely reported in the literature, with the sole clinical case from the Czech Republic (Mattioni Marchetti et al., 2023b).

Therefore, the aim of our study is to molecularly characterize an XDR carbapenemase-producing *C. freundii* isolate with high FOS MIC.

## 2 Materials and methods

### 2.1 Identification of the bacterial isolate, susceptibility determination, and detection of enzymes

Identification of the *C. freundii* strain (AK867) was confirmed by matrix-assisted laser desorption ionization–time of flight mass spectrometry (MALDI-TOF MS) with MALDI Biotyper software (Bruker Daltonics, Bremen, Germany). The production of carbapenemases (metallo-β-lactamase, OXA-48, and KPC) was assessed with the ROSCO test and with the NG-Test CARBA 5 immunochromatographic assay (NG Biotech Laboratories) (Chudejova et al., 2021). FOS MICs were evaluated using ADM and interpreted according to EUCAST clinical breakpoints v 13.0 and the new v 14.0, whereas the production of FosA-like and FosC2 enzymes was detected by the PPF test (Nakamura et al., 2014). In accordance with Nakamura in-house protocol, the PPF test requires MH agar plates added with 25 mg/L glucose-6-phosphate (G6P), confluence growth of 0.5 MacFarland solution of the isolate to investigate, one disk of FOS (50 μg), and one of FOS (50 μg) plus PPF (1 mg). The cutoff was set to a 5-mm enlargement in the inhibition zone of FOS plus PPF disk compared with the FOS disk alone (Nakamura et al., 2014; Mattioni Marchetti et al., 2023a).

### 2.2 Long-read sequencing

For genomic characterization, genomic DNA was extracted using the NucleoSpin Microbial DNA kit (Macherey-Nagel, Duren, Germany) and sheared using the Hydropore-long on Megaruptor 2 (Diagenode). Microbial multiplexing library preparation was performed without size selection according to the manufacturer’s instructions. The multiplexed library was sequenced using long-read sequencing technology using the Sequel I platform (Pacific Biosciences, Menlo Park, CA, USA) for a 10-h movie run. Assembly was performed using the “Microbial Assembly” pipeline offered by the SMRT Link v10.0. with the default settings (minimum seed coverage of 30×). Assembled sequences were annotated using the RAST (Rapid Annotation using Subsystems Technology) server (Aziz et al., 2008). *In-silico* multilocus sequence typing (MLST) of the strains and of the plasmids (pMLST) were performed when applicable. Reconstruction of the resistome, plasmidome, and virulome of the isolates was accomplished using ResFinder, PlasmidFinder, and the Virulence Factors Database (VFDB) via ABRicate (github.com/tseemann/ABRicate). BRIG v.0.95 was used to produce figures of comparison of the circular plasmids’ sequences. A linear map of chromosomal environments was created by using Easyfig (Sullivan et al., 2011) and the graphic editor Procreate (Savage Interactive, Tasmania, Australia).

### 2.3 Phylogenetic analysis

Phylogenetic relationships between AK867 and 117 global genomes, downloaded from the NCBI assembly database, including complete and draft genomes, were investigated. SNP-based phylogeny was depicted using parsnp v1.2 (Treangen et al., 2014) and using randomly GCF_029840125.1 as the reference genome. A graphic illustration of the trees was built with the Interactive Tree Of Life (iTOL) (https://itol.embl.de/). The clustering of genetic sequence was performed on the total pool of *C. freundii* ST116 by the FastBaps algorithm (Tonkin-Hill et al., 2019).

### 2.4 Conjugation/transformation assay

The conjugal transfer of *fosA* genes was tested in liquid medium using the *E. coli* J53 strain (RIF^r^) as a recipient. Transconjugants were selected on MacConkey agar plates (Scharlab, SL, Barcelona, Spain) containing rifampicin (100 mg/L) (Sigma-Aldrich, St. Louis, MO, USA), FOS (64 mg/L) (Sigma-Aldrich), and G6P (25 mg/L) (Roche). The presence of *fosA*-like genes and the plasmid content in transconjugants were further confirmed by PCR and PCR replicon typing (PBRT 2.0 kit, Diatheva), respectively (Carattoli et al., 2005).

### 2.5 Data access

The plasmid sequence of pfosA3_CFR867 has been uploaded to GenBank under the accession number CP151860–CP151866.

## 3 Results

### 3.1 Isolation and antimicrobial susceptibility profile

On 31/01/2023, a *C. freundii* (AK867) from rectal swab was collected. The sample was part of ongoing 3-year surveillance on carbapenemase-producing *Enterobacterales* (CPE) conducted locally at Modena Hospital in Italy. AK867 was isolated from a 41-year-old patient admitted in Modena Hospital, suffering from fever due to inguinal abscess by anaerobic bacteria. AK867 showed an extensively drug-resistant (XDR) phenotype, being susceptible only to colistin. FOS MIC was evaluated by ADM (FOS MIC >128 mg/L). The high FOS MIC was corroborated by the production of FosA-like enzymes, as suggested by a positive phenotypic PPF test.

### 3.2 WGS

Based on the WGS analysis, AK867 belonged to the sequence type 116 (ST116) and carried three large plasmids: an IncA (pMLST: 12) of 177,013 bp carrying the resistance genes *aph(3′)-XV*, *aadA1*, *aac(6′)-Ib-cr*, *bla*_SHV-12_, *bla*_OXA-1_, *bla*_VIM-1_, *mph(A)*, *catB2*, *catB3*, *qnrS1*, *ARR-3*, *sul1* (x2), and *dfrA14*; a multireplicon IncFIB-HI1B (pMLST IncF: F-:A-:B-; IncHI1: unknown) plasmid of 252,890 bp harboring *bla*_KPC-2_, *bla*_TEM-1A_, and *bla*_OXA-9_; and a pKPC-CAV1321 of 320,480 bp carrying *aac(3)-IIa*, *aadA2b*, *aac(6′)-Ib-cr*, *bla*_OXA-1_, *fosA3*, *ere(A)*, *cmlA1*, *catB3*, *ARR-3*, *sul1* (x2), *tet(A)*, and *dfrA19*.

### 3.3 Genomic characterization of pfosA3_CFR867

**Figure 1:**
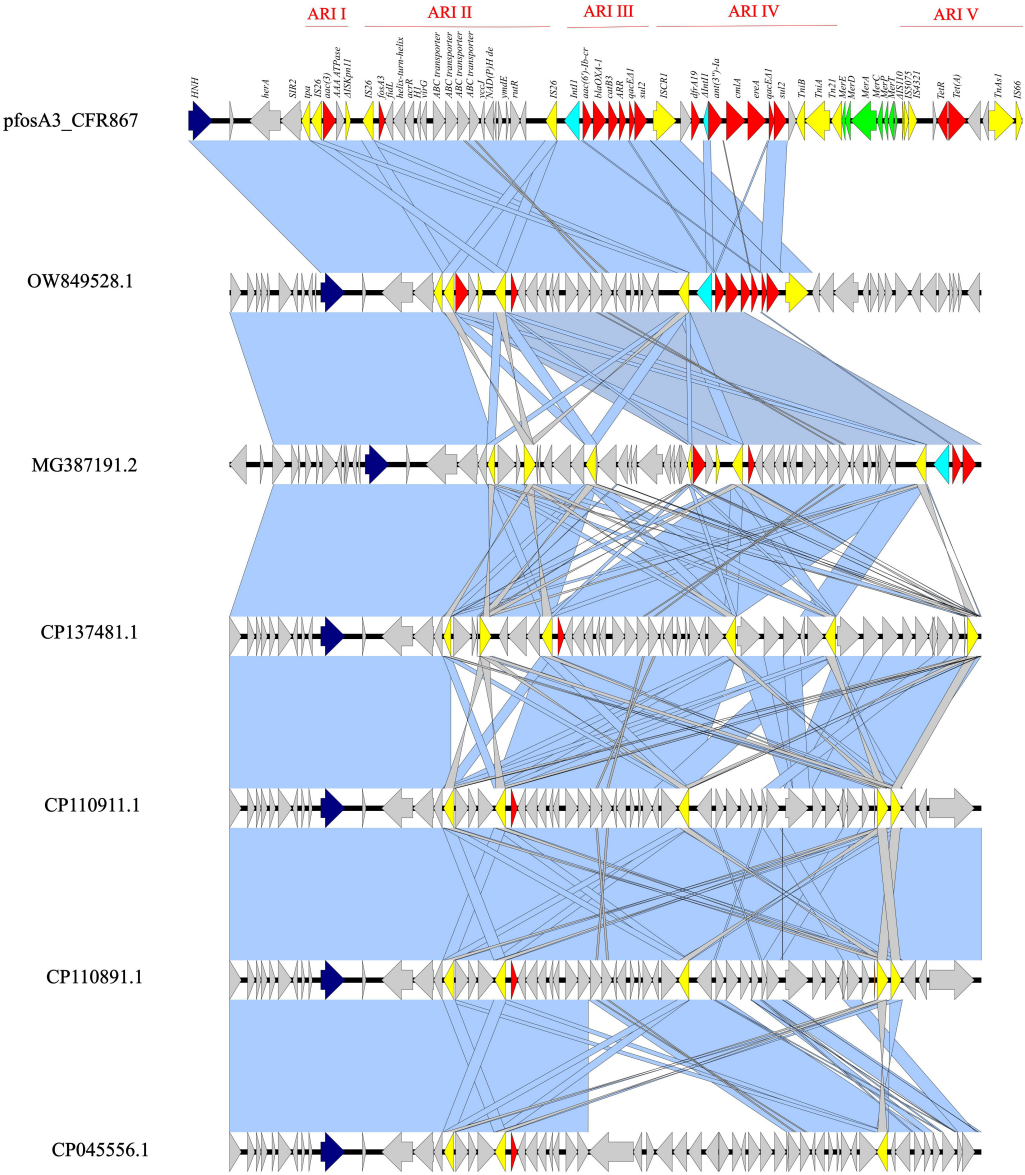
Linear map of pfosA3_CFR867 (from 241,351 bp to 296,797 bp) against OW849528.1, MG387191.2, CP137481.1, CP110911.1, CP110891.1, and СР045556.1. Dark blue: HNH endonuclease; yellow: IS; red: AMR genes; light blue: *Int1*; green: mercury system locus. Light blue shadows refer to 100% identity; gray shadows refer to identity less than 100%.

**Figure 2:**
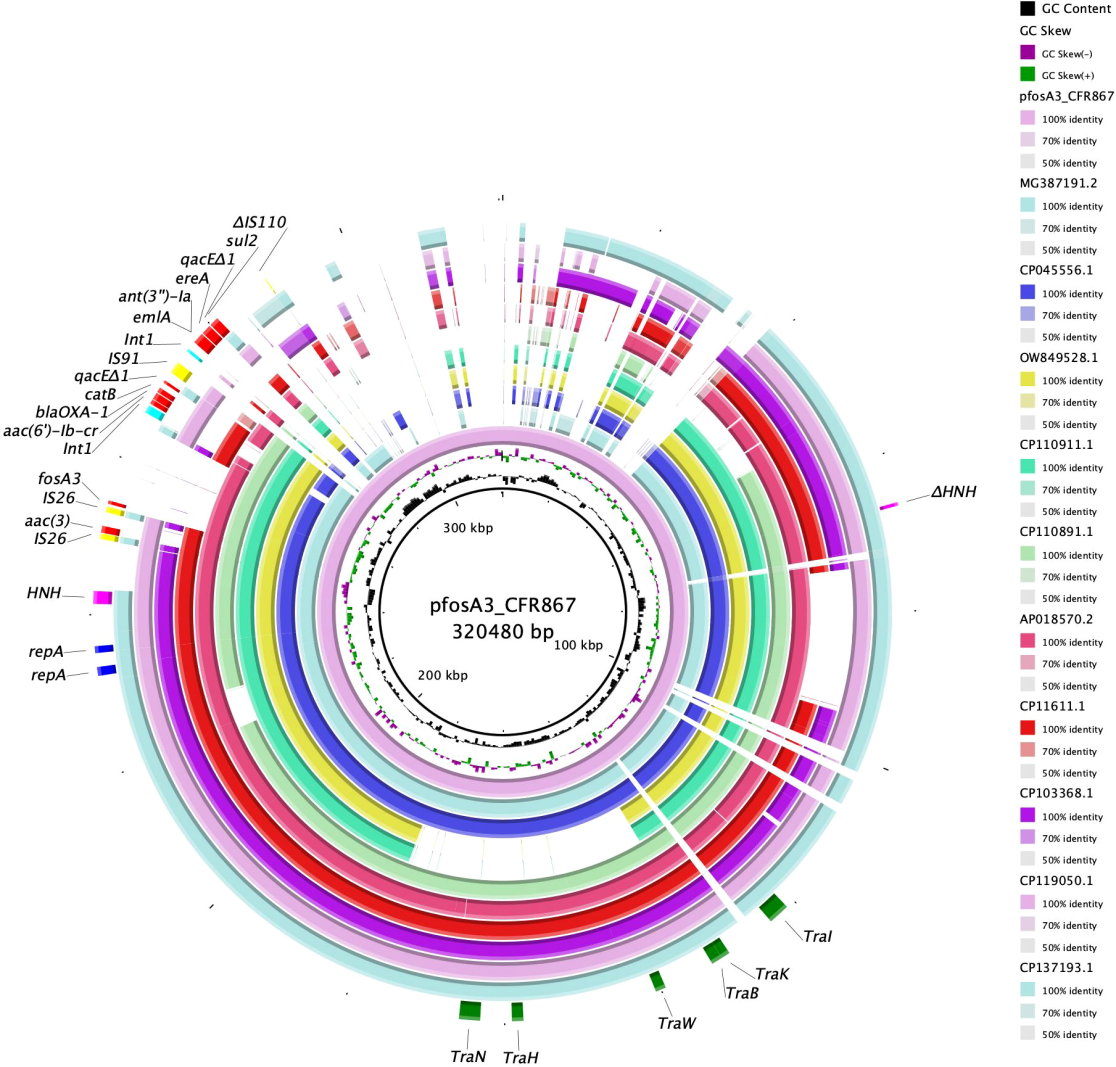
BRIG circular map of pfosA3_CFR867 against MG387191.2 (turquoise), СР045556.1 (violet), OW849528.1 (yellow), CP110911.1 (green), CP110891.1 (light green), AP018570.2 (fuchsia), CP11611.1 (red), CP103368.1 (purple), CP119050.1 (pink), and CP137193.1 (jade green). At the outer curved segments, blue, fuchsia, yellow, red, light blue and green refer to replication (repA), HNH endonuclease, IS, AMR genes, *Int1* and Tra system locus.

The pKPC-CAV1321 plasmid (pfosA3_CFR867) harbored *fosA3* as part of a large genomic island (55,446 bp), starting with an HNH endonuclease and ending with an IS*66*, and composed of five antimicrobial resistance islands (ARI): the first ARI is a IS*26*-*aac(3)*, the second a IS*26*-*fosA3*, the third *Int1*-*aac(6′)-Ib-cr*-*bla*_OXA-1_-*catB3*-*ARR-3*-*qacEΔ1*-*sul2*, the fourth a IS*CR1*-*dfrA19*-*ΔInt1*-*ant(3ʺ)-Ia*-*cmlA*-*ereA*-*qacEΔ1*-*sul2*, and the fifth is composed of ΔIS*110*-IS*5075*-IS*4321*-*tetR*-*tet(A)* (**Figure 1**). *fosA3* is inserted in a large composite transposon (12,907 bp), flanked by IS*26* at both sides, organized in IS*26*-*fosA3*-*fidL*-*helix-motif*-*acrR*-*H1*-*virG*-*ABC-transporters*-*yccJ*-*NAD(P)H*-*ymdE*-*rutR*-IS*26* (**Figure 2**). The *fosA3* transposon is entirely shared with pTEM-2262, pCFA1707-1, pCF1807-1, and pF321-1. Differently, in pS39_1, the fosA3 transposon appeared to be split, with the presence of additional *orf*s, enlarging the size of the *fosA3* transposon (**Figure 1**). These results highlighted both the conservative nature of the transposon and its ability to acquire further features.

The entire genomic island showed level of identity with other *fosA3*-harboring pKPC-CAV1312 plasmids isolated from different *C. freundii*: query 75% and ID 99.94% with pTEM-2262 (MG387191.2), from a *C. freundii* collected in China; query 63% and ID 99.98% with P1 (OW849528.1), from *C. freundii* ST22 collected in 2018 from a Spanish patient; query 63% and ID 99.96% with pCFA1707-1 (CP110911.1) and pCF1807-1 (CP110891.1), from two *C. freundii* ST107 and collected in 2017/2018 from China; query 64% and ID 99.91% with pMH17-012_4 (AP018570.2), from a *C. freundii* collected in Vietnam in 2017; query 65% and ID 99.85% with pS39_1 (CP045556.1), from a *C. freundii* ST169 collected in China in 2017; query 48% and ID 99.99% with pF321-1 (CP137481.1) from *C. portucalensis* ST252 that has been collected from urine in China in 2021 (**Figure 2**). The backbone of pfosA3_CFR867 is enriched with IS sequences, genes involved in transferability (*Tra* locus), defense system against mercury (*Mer* locus), two copies of replication genes (*rep*), and two copies of *HNH* endonucleases (**Figure 2**). These data together suggest an initial fitting of *fosA3*-harboring plasmids among *Citrobacter* spp. strains of several STs and the rearrangement ease of such plasmids. Moreover, pfosA3_CFR867 showed levels of identity with other pKPC-CAV1312 not-*fosA3*-harboring plasmids characterized from Chinese and USA *C. freundii* strains, such as CP011611.1, CP103368.1, CP119050.1, and CP137193, pointing out the conservative nature of pfosA3_CFR867 (**Figure 2**). PfosA3_CFR867 was transferable by conjugation in *E. coli* J53, shifting from FOS MIC = 0.5 mg/L to a resistance phenotype (FOS MIC >128 mg/L) in *E. coli* J53, in accordance with EUCAST clinical breakpoints v 14.0.

### 3.4 Phylogenetic analysis

**Figure 3:**
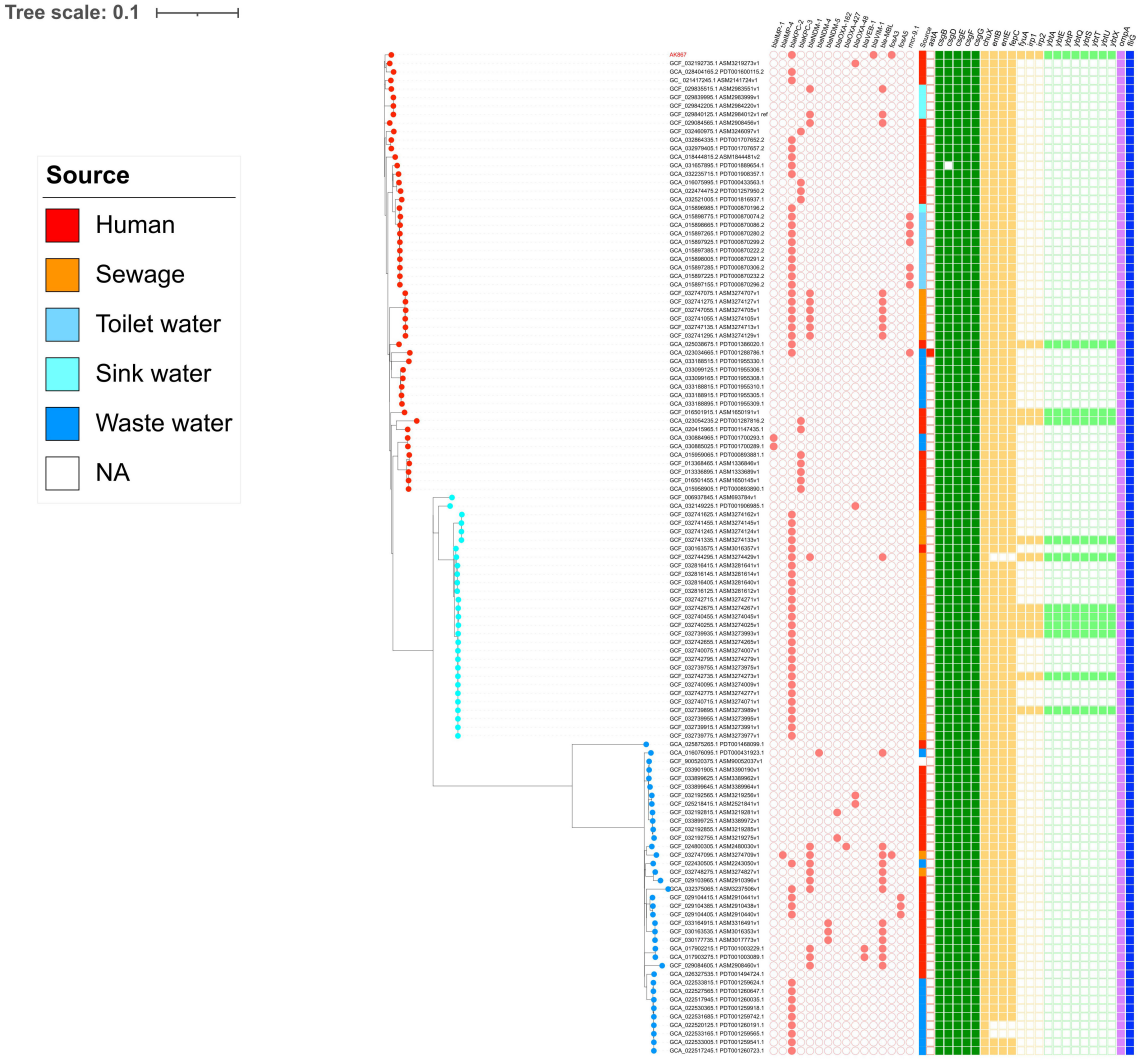
SNP-based phylogeny pictured using iTOL v6. Salmon circle grid: AMR genes. At the end of branches, red dots refer to CL3, light blue dots to CL2, and blue dots to CL1. On the squared grid (virulome), red = toxins; green = adhesion; yellow = metabolism; light green = siderophores; lilac = invasion; blue = chemotaxis.

The SNP-based phylogeny on the 118 C*. freundii* ST116 genomes downloaded from the NCBI pointed out the presence of three clades (CL1, CL2, and CL3), confirmed by the FastBaps algorithm (**Figure 3**). AK867 falls into CL3 and clustered together with GCF_032192735.1, collected in 2017 from a German patient, and GCA_028404165.2, collected in 2023 from an American patient. According to the available metadata, the three clades are circulating worldwide since 2012, with CL3 as the predominant clade. Referring to the antimicrobial resistance content, *C. freundii* ST116 revealed cluster-related resistomes, with carbapenemase KPC-2 common in the three clades. Moreover, except for AK867, the occurrence of VIM-1 carbapenemase is rare in ST116. Interestingly, the presence of *fosA3* already occurred in *C. freundii* ST116, but in CL1 from sewage sample in China (GCF_032747095.1). Moreover, another *fosA*-like gene, *fosA5*, fits in *C. freundii* ST116 CL1 as reported by three human samples collected in China (GCF_029104415.1, GCF_029104385.1, GCF_029104405.1) (**Figure 3**). Additionally, the three clusters revealed different plasmidome, with a predominance of pKPC-CAV1312 plasmids in CL2 (**Supplementary Figure S1**). pKPC-CAV1312 seemed to not easily fit in CL1 and CL3, where there is a higher incidence of IncFIB, IncFII, and IncA/C2 (**Supplementary Figure S1**). Thus, the entry and stabilization of pKPC-CAV1312 that harbor *fosA*-like genes in ST116 provide further knowledge on the real incidence of FOS resistance within *C. freundii* ST116. The presence of multireplicon IncFIB: HI1B was not reported in ST116 except for AK867. Concerning the virulome, all the three clusters shared similar virulence gene content, including adhesions (*csg* locus), metabolism (*chuX*, *entB*, *entE*, *fepC*), invasion (*ompA*), and chemotaxis (*fliG*) genes (**Figure 3**). Interestingly, AK867 showed a wider virulome, carrying the adhesion genes *fyuA*, *irp-1* and *-2*, and the locus *ybt* for siderophores (**Figure 3**).

## 4 Discussion

FosA3 is a subtype of the FosA enzyme family that, since its first report in 2010, is currently worldwide disseminated, especially in China (Hou et al., 2012; Mattioni Marchetti et al., 2023a). The major vehicles involved in dissemination are IncFII (Hou et al., 2012), followed by IncI1 (Sato et al., 2013), IncN (Liu et al., 2022), IncHI2 (Chen et al., 2021), and IncP (Hameed et al., 2022). Furthermore, it has been documented that IS*26* plays a fundamental role in antimicrobial resistance genes (AMR) transposition and dissemination among *Enterobacterales* (Lv et al., 2020). Here, we depicted the genomic organization of a novel *fosA3*-harboring transposon on a pKPC-CAV1312 plasmids. As suggested by the available genomes on NCBI, pKPC-CAV1312 plasmids are likely to promote the entry and the subsequent fitting of *fosA3* in *Citrobacter* spp. Interestingly, pfosA3_CFR867 did not show a perfect identity with others *fosA3*-mediated pKPC-CAV1312 plasmids, suggesting consistent rearrangements in the plasmid backbone structure. The plasmid-mediated *fosA3* is generally organized in a composite transposon of 4 kb in size, consisting in two IS*26* elements with the same orientation, flanking the cassette *fosA3-orf1-orf2-Δorf3* (Wachino et al., 2010). In the present study, *fosA3* was included in a large composite transposon of >12 kb that contained several *orf*s. The occurrence of *fosA3* in large transposon, with the ability to carry different genes, and combined with the transposition potential of IS*26*, poses a further challenge in containing the spread of such emergent antibiotic-resistant strains at a global level.

The HNH endonucleases are a group of homing endonucleases that can act as selfish genetic elements, like transposons, breaking double-strand DNA and allowing the acquisition of functional attributes to the host cell, such as AMR genes (Edgell, 2009). The association between *fosA*-like genes and HNH endonucleases has already been pointed out in literature, speculating an undefined role of HNH in the dissemination of *fosA* genes, in absence of any surrounding insertion elements, within *Citrobacter* spp (Li et al., 2020; Mattioni Marchetti et al., 2023b). The pfosA3_CFR867 contained an HNH endonuclease, at a 6,530-bp distance from the *fosA3* transposon.

In the present study, AK867 showed an XDR profile due to the co-presence of clinically relevant carbapenemases KPC-2 and VIM-1. In this prospective, the resistance to carbapenems and high FOS MIC in *C. freundii* could represent a novel menace and reduce the current antimicrobial *armamentarium*. This possibility is also strengthened by the easy transfer of such gene into the *E. coli* recipient, inducing an increase in MIC values beyond the current EUCAST breakpoints (resistance category with MIC >8 mg/L).

EUCAST clinical breakpoints for FOS underwent on a recent revision, with FOS cutoffs applicable on *E. coli* only and not recommending the use of FOS for other *Enterobacterales* than *E. coli*. However, FOS still represents a valid option in the treatment of urinary tract infection by ESBL-producing *E. coli* and *C. freundii* (Bielen and Likic, 2019). Moreover, FOS is also recognized as a valid option in combination therapy against several MDR Gram-negative infections, due to its relevant synergistic effect (Antonello et al., 2020). Based on the previous EUCAST breakpoints, *C. freundii* strains maintain high susceptibility levels to FOS (average MIC = 4 mg/L), but an eventual stabilization of transferable FosA enzymes may mark a turning point in the evolution of antibiotic resistance in *C. freundii* (Bielen et al., 2018).

In fact, cases of FosA-like enzymes in uncommon pathogens, as *C. freundii*, are slowly emerging in the literature (Chen et al., 2021; Mattioni Marchetti et al., 2023a; Mattioni Marchetti et al., 2023b). For this reason, despite the EUCAST revision, FosA enzyme detection and related transferability should be assessed even in low-risk pathogens, in order to track the transmission routes from these pathogens to clinically relevant clones, such as *E. coli* ST131 (Chudejova et al., 2024). Doubtless, large-scale surveillance on FOS-resistance profiles among *Enterobacterales* are demanding due to the lack of rapid kit as reliable as the reference ADM method. However, the coexistence of carbapenemases and FosA-like enzymes requires additional effort in clinical surveillance programs (Nakamura et al., 2014).

In conclusion, the incidence of FOS resistance is increasing globally among *Enterobacterales*, reaching and fitting even in low-risk pathogens, such as *C. freundii*. Together with carbapenem resistance, FOS resistance strains pose clinical challenges that, to be addressed, required dedicated surveillance programs and alternative rapid detection methods.

## Data availability statement

The datasets presented in this study can be found in online repositories. The names of the repository/repositories and accession number(s) can be found in the article/**Supplementary Material**.

## Ethics statement

The study was designed and conducted in accordance with the Helsinki Declaration. The work described herein is molecular study performed on bacterial isolate from human sample that were obtained as part of routine hospital care and used anonymously. Consent to participate was not required, as samples were collected as part of standard patient care. The studies were conducted in accordance with the local legislation and institutional requirements. The human samples used in this study were acquired from a by-product of routine care or industry. Written informed consent to participate in this study was not required from the participants or the participants’ legal guardians/next of kin in accordance with the national legislation and the institutional requirements.

## Author contributions

VM: Conceptualization, Investigation, Writing – original draft, Writing – review & editing. IV: Formal analysis, Writing – review & editing. TC: Data curation, Formal analysis, Writing – review & editing. MM: Data curation, Writing – review & editing. RM: Validation, Writing – review & editing. IB: Funding acquisition, Supervision, Validation, Writing – review & editing.

## Supplementary material

Supplementary Figure 1 Heatmap representation of plasmids content among 118 ST116 *C. freundii*. Light blue dots= absence; red dots= presence. Blue labels=CL1, light blue labels=CL2, red labels=CL3.
