# Supplementary figures and images for: FosA3 emerging in clinical carbapenemase-producing C. freundii
Source: Front Cell Infect Microbiol. 2024 Aug 6;14:1447933. doi: 10.3389/fcimb.2024.1447933 (PMC11378647; doi:10.3389/fcimb.2024.1447933)

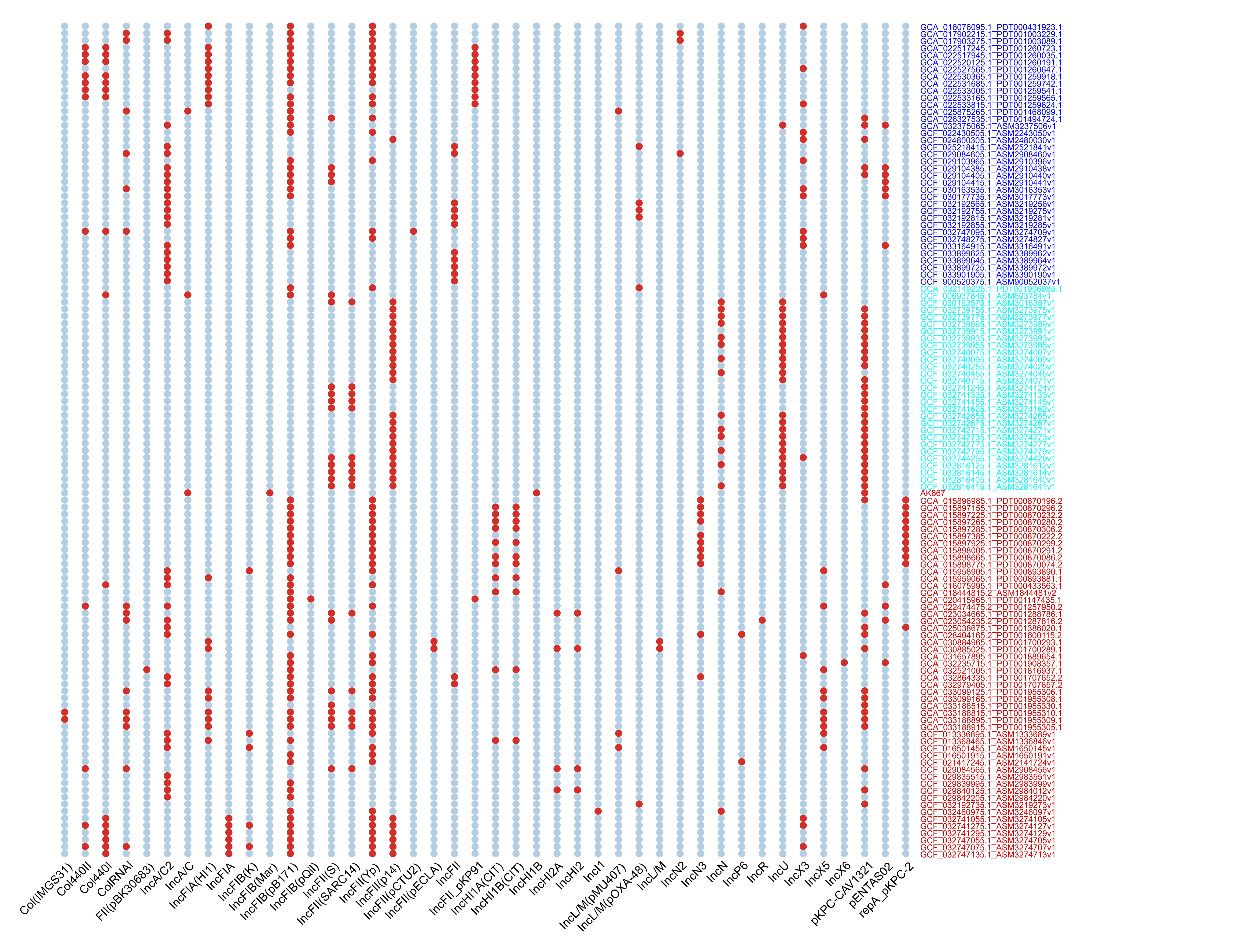

Supplement: Supplementary Figure 1 — Heatmap representation of plasmids content among 118 ST116 C. freundii. Light blue dots= absence; red dots= presence. Blue labels=CL1, light blue labels=CL2, red labels=CL3. [file Image1.png]
